# Drone thermal imaging and benthic time-series analysis show dynamic spatial and temporal delivery of submarine groundwater discharge on reef ecosystems

**DOI:** 10.1371/journal.pone.0333712

**Published:** 2025-10-03

**Authors:** Sophia G. Zummo, Lucian Himes, Florybeth F. La Valle

**Affiliations:** 1 Marine Biology Research Division, Scripps Institution of Oceanography, University of California San Diego, La Jolla, California, United States of America; 2 Natural Science Division, Pepperdine University, Malibu, California, United States of America; National Taiwan Ocean University, TAIWAN

## Abstract

Submarine groundwater discharge (SGD) introduces nutrient-rich, low-salinity water into coastal ecosystems, significantly altering reef biogeochemistry. At Black Point, Oʻahu, we employed a novel, two-pronged approach that integrates a cost-effective small unmanned aerial system equipped with a thermal infrared (sUAS-TIR) sensor and high-resolution benthic salinity time series to resolve previously unobserved, fine-scale patterns of SGD delivery. sUAS-based sampling overcomes key limitations of prior SGD mapping methods, such as labor-intensive and spatially constrained in situ sampling, by enabling rapid deployment, real-time visualization of mixing dynamics, and high-resolution imagery. Unlike previous studies that relied on customized systems that required independent sensor integration, our methods used a cost-effective, fully integrated sUAS-TIR platform that required no additional modification. Our results show that localized hydrodynamics strongly modulate groundwater delivery, creating spatially heterogeneous patterns of delayed transport, recirculation and retention (i.e., pooling), and offshore dispersal. Thermal imagery revealed persistent surface plumes in regions of reduced circulation, while sequential orthomosaics and benthic salinity time series captured the temporal progression of groundwater movement across the reef. These findings highlight complex SGD delivery patterns that contribute to ecological vulnerability, particularly in areas experiencing prolonged exposure to nutrient-rich, low-salinity waters. Such dynamics underscore the need to consider both spatial and temporal variability when evaluating SGD’s ecological impacts. Our two-pronged approach offers a valuable tool to (1) identify reef zones that may be disproportionately affected by point-sources of land-based pollution, and (2) elucidate the mechanisms driving groundwater transport in coral reef environments. By offering a cost-effective, scalable, and operationally flexible framework, this methodology advances the management of groundwater-impacted ecosystems and enhances our ability to assess the ecological implications of dynamic SGD delivery.

## Introduction

Submarine groundwater discharge (SGD) is the flow of terrestrial freshwater and recirculated seawater into the coastal ocean, transporting dissolved nutrients, organic matter, and trace elements into nearshore ecosystems [1,2]. Its chemical composition is geographically unique, due to water residence times, precipitation rates, recirculation, land use practices upstream, and biogeochemical processes within the sub-estuarine aquifer [2–4]. SGD can significantly alter the biogeochemistry of nearshore reefs [5–7], elevating nutrient concentrations, lowering salinity levels, and altering the carbonate chemistry of the system [8–11], which can impact local marine biota [3,12,13] and influence benthic community structure [14–17].

On high volcanic islands, SGD is primarily freshwater, with salinity levels as low as 5 ppt and nutrient concentrations reaching three orders of magnitude above background levels, depending on terrestrial land use [3,6,18]. Additionally, SGD delivery is tidally modulated with fluxes peaking at low tide [19–21]. Thus, benthic organisms on SGD-impacted reefs must tolerate rapid, tidally driven fluctuations in salinity and nutrient availability [14–16]. Marine primary producers exhibit species-specific tolerance ranges, with algal uptake rates and productivity influenced by nutrient concentrations [3,22,23] and osmotic regulation dependent on salinity [24–26]. Consequently, SGD creates environmental conditions that selectively favor species capable of withstanding wide fluctuations in nutrient and salinity levels. Recent studies estimate that approximately 14% of global coral reefs are vulnerable to eutrophication driven by SGD [27], emphasizing the need to better map, quantify, and understand SGD distribution and its ecological consequences.

Despite widespread recognition of its ecological significance, tracking and characterizing SGD remains challenging due to its dynamic, patchy nature. Tidally driven SGD is spatially and temporally dynamic, with fluxes typically peaking at low tide due to reduced offshore hydrostatic pressure [19–21]. This cyclical delivery of SGD makes it difficult to track and quantify the groundwater, as the biogeochemistry of SGD is highly variable on small temporal scales (i.e., hours) and spatial scales (i.e., meters) [28,29]. While traditional approaches relying on geochemical tracers (i.e., radium, radon, methane, silica, hydrogen, and oxygen stable isotopes of water) enable site-specific SGD detection [29–31], they often require large volumes of seawater to be collected, making these methods laborious and difficult to execute over large areas [2,32]. Furthermore, these methodologies can have high uncertainties due to the difficulty of calculating groundwater endmembers [8,33,34].

More recently, thermal infrared (TIR) sensing has emerged as an effective proxy for detecting SGD due to the density differences between fresh and saltwater, resulting in the cooler freshwater remaining on the surface [35–37]. Although TIR mapping from satellites [36,38–41] and manned aircrafts [37,42–44] has been successful in capturing SGD on large spatial scales (i.e., km), these platforms lack the flexibility and resolution needed to capture fine-scale variability critical for understanding nearshore SGD delivery and mixing. Additionally, the biogeochemistry of SGD is site specific, dependent on land-use practices upstream [3,4] as well as aquifer source [2,6]. Hence, reefs as close as a couple kilometers can have distinct nutrient loadings due to the biogeochemical differences experienced by different aquifers [5,6,13]. As a result, it is critical to study SGD at the local scale, as both its biogeochemical composition and resulting ecological effects can vary substantially between sites.

Small unmanned aerial systems (sUAS) equipped with TIR sensors offer a high-resolution, operationally flexible alternative for mapping SGD distribution on smaller, localized scales. Previous studies demonstrated the feasibility of custom sUAS-TIR systems to delineate groundwater plumes, though many relied on expensive, customized sensor integration [32,45–47]. Building on this progress, our study highlights the functionality of a cost-effective, sUAS-TIR system that requires no modification, allowing for rapid deployment, high spatial resolution (~15–25 cm), and real-time surface thermal visualization at a lower cost. To comply with the Federal Aviation Administration (FAA) 120-meter altitude limit for drone operations, we used thermal orthomosaics to capture SGD distribution. This approach not only enables coverage of larger spatial areas, but also preserves high spatial resolution, which would otherwise decrease with increasing flight altitude.

Understanding the ecological consequences of SGD depends not only on quantifying the presence and concentration of low-salinity, nutrient-rich groundwater, but also elucidating the local mixing conditions that govern SGD influence. These dynamics are shaped in part by the physical properties of SGD: being fresher and less dense than ambient seawater, it remains buoyant near the surface before mixing vertically [35,36]. As a result, surface and benthic regions may experience distinct spatial and temporal patterns of SGD exposure. While tidal variability plays a role in modulating SGD delivery, reduced mixing and prolonged residence times can disproportionately affect certain reef zones, leading to localized nutrient loading and physiological stress. Limited circulation has been linked to increased mortality [48,49], reduced growth [13,50], and heightened stress and disease in corals [51,52]. Moreover, algal growth and survivorship are strongly shaped by water motion, which facilitates nutrient uptake and waste removal, but can also impose physical stress [53,54]. Thus, circulation dynamics are a critical, but often overlooked, factor driving SGD influence across coral reefs.

To resolve these complex mixing dynamics, we designed a two-pronged approach that combines real-time thermal visualization with high-resolution benthic salinity time-series. This integrated method captures conditions at the surface and benthos simultaneously, enabling the detection of fine-scale circulation patterns (i.e., reduced flow, groundwater retention, plume dispersion) that traditional stationary methods cannot resolve alone. Surface imagery captures the spatial distribution of SGD, whereas in situ salinity data provide temporal resolution and confirm groundwater exposure at the benthos. Here, we applied this approach to Black Point, Oʻahu (Hawaiʻi), where previous geochemical and in situ studies identified a localized SGD seep [5,6,19], but lacked spatial coverage of surface plume dynamics. Specifically, we assessed whether sUAS-derived surface thermal patterns aligned with benthic salinity signatures and evaluated their potential to capture aspects of groundwater distribution that are not resolved by solely monitoring the benthos. Our findings highlight the value of this two-pronged approach for (1) identifying reef zones that may be disproportionately affected by point-sources of land-based pollution, and (2) improving our understanding of the physical mechanisms driving groundwater movement in coral reef environments across various spatial and temporal scales.

## Methods

### Study site description

The Maunalua region along the southeast coast of O‘ahu, Hawai‘i has experienced dramatic development and population increase over the last 100 years, affecting the water quality of the nine watersheds that feed into the bay [55]. SGD is the primary source of terrestrial water in Maunalua Bay and high concentration of wastewater throughout the region has led to increased nutrient concentrations in the groundwater [56]. Thus, Maunalua Bay is a model system to study anthropogenically influenced SGD.

**Fig 1:**
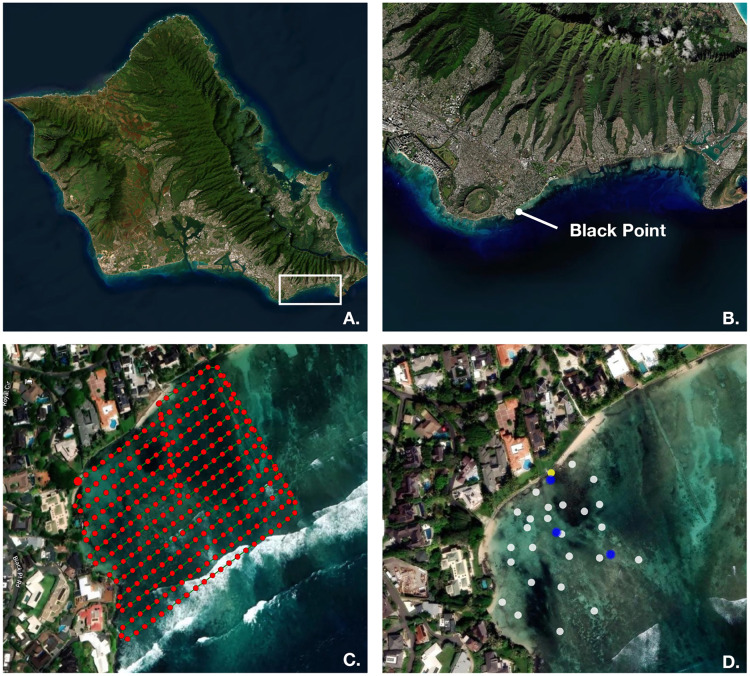
Study site maps. (A) Map of O‘ahu with Maunalua Bay outlined in a white box. (B) Close up of the Maunalua Bay shoreline; Black Point reef is located on the western edge of the bay near Diamond Head. (C) Drone flight path visualized in PIX4Dmapper; each red circle denotes where a TIR image was taken. (D) The localized SGD seep is shown in yellow (21.2586°N, −157.7899°W), where a benthic salinity sensor was deployed. Additional benthic sensor locations are shown in white. In situ validation data was collected at each blue label and at the seep. Created by authors using base maps from Esri. Content is the intellectual property of Esri and is used herein with permission. Copyright © 2025 Esri and its licensors. All rights reserved. Sources for basemaps: Resource Mapping Hawaii; Esri; Maxar/DigitalGlobe; Earthstar Geographics; CNES/Airbus DS; USDA FSA; USGS; Aerogrid; IGN; IGP; and the GIS User Community.

Our study site, Black Point, is a shallow reef flat (<1m deep at mean low water, [13]) along the westernmost end of Maunalua Bay (Fig 1). Black Point has one localized SGD source close to the shoreline (21.2586°N, −157.7899°W, [5]) and is composed of patches of continuous carbonate shelves and isolated boulders surrounded by sand [13]. Species diversity at Black Point decreases with increasing SGD exposure; the site has low coral cover and is dominated by fleshy algae [16].

### In situ validation sampling

A DJI Mavic 3T Enterprise drone with a three-camera payload, including an Uncooled VOx Microbolometer thermal camera with a resolution of 640 × 512 and a 61° field of view, was used to acquire aerial TIR imagery. This drone was selected because it is a fully integrated TIR-sUAS system, requiring no additional modification. The infrared temperature measurement accuracy of this Microbolometer is ± 2 °C [57]. While this level of accuracy limits the system’s suitability for precise surface temperature measurements, it does not compromise its utility for detecting relative thermal contrasts. The consistency of thermal patterns across repeated surveys, as shown in orthomosaics, supports the use of these relative contrasts as a proxy for SGD distribution. All pilots possessed a Part 107 Remote Pilot Certificate, and no additional permits were required for night operations. Anti-collision lights visible from three statute miles were attached to the drone and it remained in visual line of sight at all times [58].

Validation data was collected repeatedly over four nights during low tide conditions at four biogeochemical zones characterized by SGD distribution that were defined in a previous study (seep, transition, diffuse, and ambient, [5]). Flight duration was constrained by battery life, with each survey requiring approximately 40 minutes and typically consisting of 3 flights.

A YSI ProQuatro was used to continuously sample temperature and salinity in situ synchronously with drone thermal images. The drone was flown at 25 ± 5 m altitude from the surface of the water and samples were collected from the drone and YSI every 2 seconds for a minute. Thermal images were analyzed using the DJI Thermal Analysis tool; the average temperature in an approximate 15 × 15 cm area was extracted manually for every picture (n = 251) in areas upstream of and contiguous to the YSI.

Immediately following YSI sampling, water samples were collected at the same locations where the temperature validation data was taken. Water samples were filtered with 0.2 µm GF/F filters and frozen until analysis. Inorganic nutrient samples were analyzed by the Oceanographic Data Facility at Scripps Institution of Oceanography on a Seal Analytical continuous-flow AutoAnalyzer 3 (AA3) following methods described by Atlas et al. [59], Hager et al. [60], and Gordon et al. [61].

### Orthomosaic production

Previous research documented SGD biogeochemical signals (i.e., elevated nutrient concentrations, decreased salinity, etc.) within 200m of the SGD seep at Black Point [5]. Due to the 120-meter maximum height for drones imposed by the FAA, it was necessary to produce orthomosaic maps as the SGD plume could not be captured in a single image while following FAA guidelines.

A flight path encompassing 48,020 m^2^ was created in the Enterprise flight interface DJI Pilot 2 and included the coastline to improve the stitching ability of the mapping software (Fig 1). Approximately 300 photos were taken over the span of ~12 minutes to produce an orthomosaic with an 85% overlap and sidelap ratio and a ground sampling distance of 15.73 cm/pixel. The drone was programmed to take TIR images every 12 meters, ensuring that overlap was consistent between images, regardless of speed changes from wind gusts or turns.

All TIR data was collected during the night to eliminate solar heating artifacts [32,37] and in partly cloudy to no cloud conditions. Low tides (−0.07–0.04 m) were targeted for orthomosaics, and a time series of SGD distribution was examined over the course of an incoming tide, beginning approximately 38 minutes after the observed low tide and sampling every 30 minutes after until the next high tide.

Individual mapping images were converted from jpegs into tiff files using the open-source program DJI Image Processor.exe [62] as the mapping software requires tiff files. Orthomosaics were generated in the software PIX4Dmapper Desktop v.4.9 using the thermal imagery workflow and saved as a grayscale index tiff file. Tiff files were converted to rasters in R and reprojected onto the correct coordinate system using the packages raster, tiff, sp, terra, and matrix. Rasters were then converted into data frames and overlaid on Esri imagery using the leaflet package. Additionally, the surface area of the SGD plume was calculated in R by manually delineating the plume boundary and taking the area of the resulting polygon.

### Statistical analyses

Linear regressions were performed between YSI salinity and inorganic nutrients. Model II regressions using the major axis (MA) method evaluated the associations between YSI-measured temperatures and average drone-derived temperatures, as well as YSI-measured temperatures and YSI-measured salinity (R package lmodel2). Temporal lags in salinity changes between benthic sensors were assessed using cross-correlation analyses, with one lag unit corresponding to a six-minute interval. The cross-correlation function (CCF) in R identifies whether variations in one signal consistently precede or follow variations in another, thereby revealing potential time delays and directional influences between spatially explicit time series. The estimated lag time was identified as the lag corresponding to the maximum cross-correlation coefficient. Following the approach of Mallast and Siebert [63], we used autocorrelation functions to assess spatial and temporal variations in SGD distribution. Significant peaks exceeding the 95% confidence interval indicated the persistence of salinity patterns over time at individual sensor locations.

## Results

### Relationships between inorganic nutrients and salinity

There were significant linear relationships between YSI salinity and inorganic nutrients (i.e., nitrate, phosphate, silicate, and nitrite) of water samples collected across the SGD plume (n = 28 paired samples, Table 1). The observed trends are characteristic of conservative mixing, where SGD enters with low salinity (~5 ppt) and elevated nutrient concentrations and is increasingly diluted as it mixes with surrounding ambient coastal waters. Previous studies reported similar linear relationships at Black Point [6,22]. Given that these inorganic nutrients covary significantly on this reef and that SGD is the primary source of both freshwater and nutrients, salinity serves as a proxy for nutrient concentrations and a useful tracer for SGD contribution.

**Table 1:** Summary statistics of (1) linear regression models (df = 26) of salinity and inorganic nutrients and (2) Model II regressions evaluating the associations between YSI-measured temperatures and average drone-derived temperatures, as well as YSI temperatures and YSI-measured salinity.

Test	Summary Statistics		
	Line of best-fit	R^2^	p-value
**NO**_**3**_ **vs salinity**	−5.01 × salinity + 183.43	0.9352	2x10^-16^
**PO**_**4**_ **vs salinity**	−0.084 × salinity + 3.30	0.8850	1.01x10^-13^
**SiO**_**4**_^**4-**^ **vs salinity**	−21.20 × salinity + 777.08	0.9356	2x10^-16^
**NO**_**2**_ **vs salinity**	−0.005 × salinity + 0.28	0.1658	0.031527
**Avg drone temperature** **vs YSI temperature**	0.92 x YSI temperature + 2.3	0.524	0.017
**YSI salinity vs** **YSI temperature**	47 x YSI temperature – 1100	0.549	0.001

### Relationships between drone temperature and in situ values

**Fig 2:**
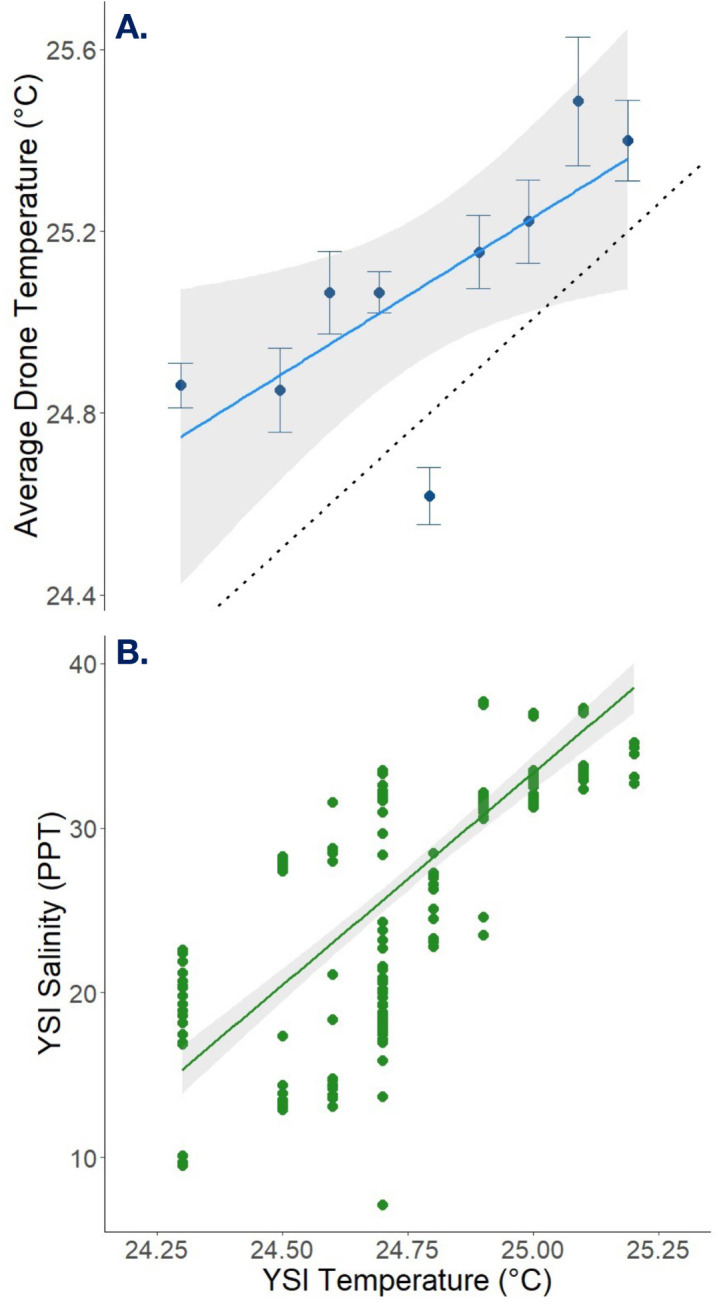
Model II regressions using the MA method revealed strong associations between (A.) YSI-measured temperatures and average drone-derived temperatures and (B.) YSI-measured temperatures and YSI-measured salinity.

Paired temperature values (n = 251) were used to assess the relationship between YSI water temperatures and drone-derived surface water temperatures (Fig 2A). Drone temperatures were averaged for each corresponding YSI temperature, and a Model II regression using the major axis (MA) indicated strong agreement between methods (p = 0.017; Table 1). While the drone-derived temperatures were consistently higher than in situ YSI measurements, the results show that drone imagery reliably captures relative changes in temperature. Although not suitable for absolute temperature detection, the drone data effectively reflects spatial and temporal variations in surface water temperature that are associated with SGD plumes. A similar observation has been reported, where UAV-mounted thermal sensors exhibited a warm bias in absolute temperature readings, yet still revealed reliable spatial patterns of localized spring water inputs in river systems [64].

A second Model II regression using the MA method revealed a strong relationship between YSI-measured temperatures and YSI-measured salinity values (n = 251 paired samples; p = 0.001; Table 1; Fig 2B). This relationship is consistent with previous observations indicating that SGD at Black Point is colder and fresher than surrounding ambient waters [6]. Thus, spatial variations in surface water temperature can serve as a proxy for SGD distribution.

### SGD plume structure and stability

**Fig 3:**
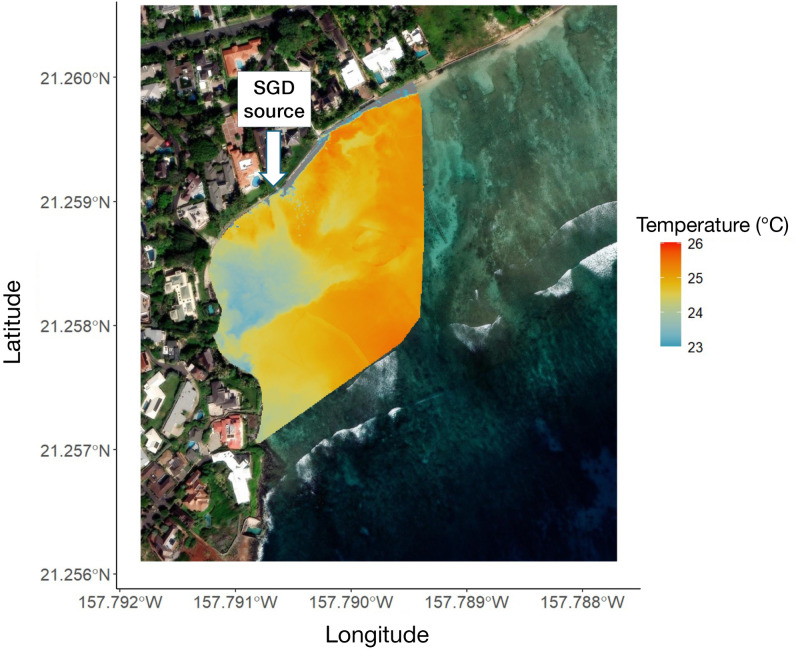
Distribution of SGD at Black Point mapped using sUAS thermal infrared imagery 72–84 minutes after the local low tide (estimated tidal height: 0.04 m; flight time and date: 5:30–5:42, 13 May 2024). The localized SGD seep near the shoreline (21.2586°N, 157.7899°W) is indicated by the arrow. Cooler temperatures associated with SGD are visible extending offshore (~100m), showing the spatial distribution and persistence of the plume under low tidal conditions. Created by authors using base maps from Esri. Content is the intellectual property of Esri and is used herein with permission. Copyright © 2025 Esri and its licensors. All rights reserved. Sources for basemaps: Resource Mapping Hawaii; Esri; Maxar/DigitalGlobe; Earthstar Geographics; CNES/Airbus DS; USDA FSA; USGS; Aerogrid; IGN; IGP; and the GIS User Community.

The distribution of SGD during low tide conditions at Black Point on 13 May 2024 is illustrated in Fig 3. Despite a relatively small thermal contrast (~2–3 °C), the sUAS-TIR imagery successfully captured the cooler SGD plume extending ~100 m offshore, with an approximate surface area of 8,334 m^2^. While previous studies identified a localized SGD seep at Black Point (21.2586°N, −157.7899°W) using geochemical and in situ methods [5,6,19], this study presents the first sUAs-derived thermal visualization of SGD distribution at this site, revealing pronounced surface retention of the groundwater plume. SGD appears to recirculate and accumulate along the western shoreline of the reef (21.25795°N, −157.79101°W); we define this process as SGD pooling: the localized, short-term retention of discharged groundwater due to reduced circulation. Repeated surveys conducted between May and June 2024 showed a plume with consistent spatial position and offshore dispersal at low tide, underscoring persistent discharge dynamics, stable plume behavior across short temporal scales (i.e., weeks to months), and consistent orthomosaic production, despite variable wind conditions (S1 Fig; S1 Table).

**Fig 4:**
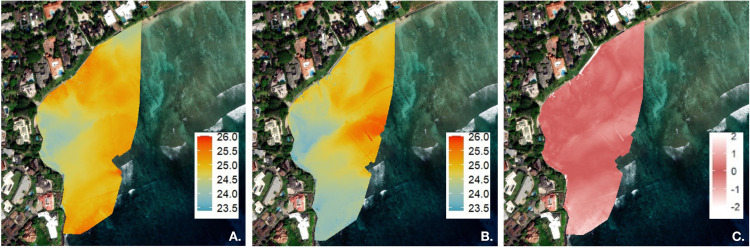
Consecutive orthomosaics were collected on June 11, 2024 (A: 04:08–04:20; B: 04:26–04:38). **(C)** The difference in surface temperature between the two orthomosaics **(A-B)**. The greatest temperature differences were observed offshore, while minimal variability was detected within the plume. Although mapping the full study area required approximately 12 minutes, plume orientation and structure remained stable throughout the mapping period. Created by authors using base maps from Esri. Content is the intellectual property of Esri and is used herein with permission. Copyright © 2025 Esri and its licensors. All rights reserved. Sources for basemaps: Resource Mapping Hawaii; Esri; Maxar/DigitalGlobe; Earthstar Geographics; CNES/Airbus DS; USDA FSA; USGS; Aerogrid; IGN; IGP; and the GIS User Community.

Consecutive orthomosaics were collected on June 11, 2024 (Fig 4, A: 04:08–04:20; B: 04:26–04:38) to evaluate and qualitatively present plume stability. Although mapping the full study area required approximately 12 minutes, the plume’s orientation and structure remained consistent throughout the mapping period. The greatest temperature differences were observed offshore while minimal temperature variability was detected within the plume (Fig 4C). This is consistent with benthic salinity autocorrelation patterns, which show that salinity at the seep remained autocorrelated for approximately 3.3 hours, indicating relatively stable conditions over the time period (S2C Fig). Together, these results demonstrate that over hourly timescales plume structure is preserved.

### Temporal patterns of SGD delivery, pooling, and offshore transport

**Fig 5:**
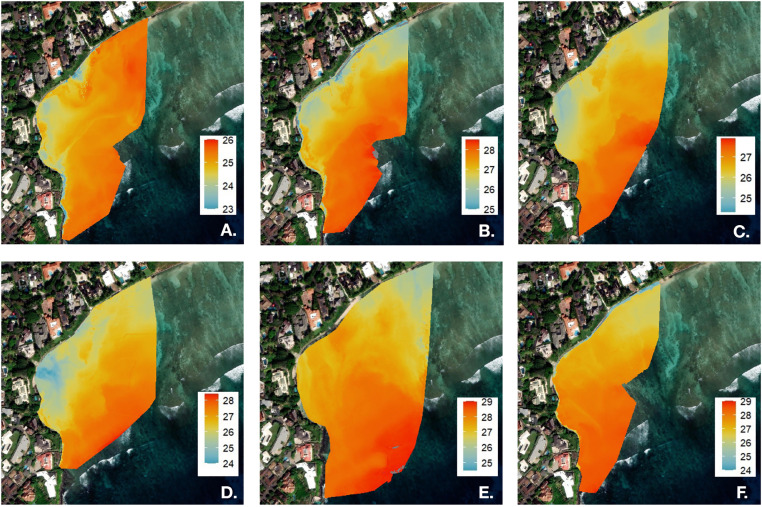
SGD distribution was examined over the course of an outgoing tide, beginning approximately 38 minutes after the observed low tide on March 1, 2025 (A: 23:44-23:58, estimated tidal height (eth): 0.009 m; B: 00:46-01:00, eth: 0.11 m; C: 01:15-01:29, eth: 0.17 m, D: 1:45-1:59, eth: 0.24 m, E: 2:45-2:59, eth 0.37 m, F: 3:47-4:01, eth: 0.48 m). SGD pools in the western region of the reef where it accumulates during the ebb tide and disperses offshore during flood tide conditions. Created by authors using base maps from Esri. Content is the intellectual property of Esri and is used herein with permission. Copyright © 2025 Esri and its licensors. All rights reserved. Sources for basemaps: Resource Mapping Hawaii; Esri; Maxar/DigitalGlobe; Earthstar Geographics; CNES/Airbus DS; USDA FSA; USGS; Aerogrid; IGN; IGP; and the GIS User Community.

SGD distribution was examined over the course of an outgoing tide, beginning approximately 38 minutes after the observed low tide (Fig 5A; estimated tidal height: 0.009 m). Thermal imagery was collected at 30-minute intervals, with the final map acquired approximately 5 hours after low tide, corresponding to an estimated tidal height of 0.48 m (Fig 5F). Consistent with previous studies reporting peak SGD flux during an ebb tide [19–21], our high-resolution orthomosaics also captured the gradual accumulation of groundwater in the reef’s western region following low tide. In certain areas, this recirculating retention (i.e., pooling) delays offshore transport and prolongs SGD exposure beyond the ebb tide. Our observations indicate that pooled groundwater is gradually dispersed offshore during flood tide conditions, approximately 4 hours after low tide (Fig 5E; estimated tidal height: 0.37 m).

**Fig 6:**
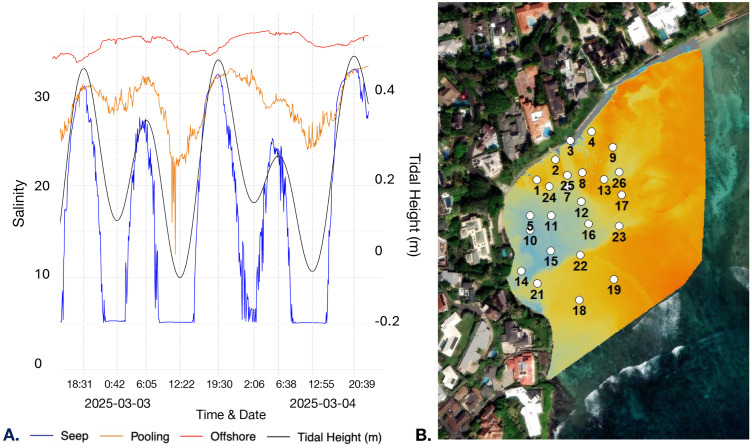
Benthic salinity dynamics across the reef exhibited distinct temporal relationships with tidal height. **(A)** Time series of benthic salinity at sensors 3 (seep, blue), 14 (pooling area, orange), and 19 (offshore, red), with overlaid tidal height (meters). Time stamps correspond to low and high tides. **(B)** Sensor grid plotted on a low-tide thermal orthomosaic. Created by authors using base maps from Esri. Content is the intellectual property of Esri and is used herein with permission. Copyright © 2025 Esri and its licensors. All rights reserved. Sources for basemaps: Resource Mapping Hawaii; Esri; Maxar/DigitalGlobe; Earthstar Geographics; CNES/Airbus DS; USDA FSA; USGS; Aerogrid; IGN; IGP; and the GIS User Community.

Benthic salinity dynamics across the reef exhibited distinct temporal relationships with tidal height (Fig 6A), revealing a clear temporal progression of (1) SGD delivery at the localized seep, (2) followed by groundwater pooling, and (3) offshore transport. Salinity minima at the seep preceded localized low tides, while the falling tide preceded decreases in salinity at sensor 14, indicating SGD pooling. At sensor 19, salinity declined prior to high tide, reflecting the gradual offshore transport of pooled groundwater via tidal advection. These patterns are also visible in sequential thermal orthomosaics (Fig 5), which show cooler groundwater pooling along the western shoreline before gradually dispersing offshore.

While thermal imagery showed SGD expression encompassing other sensor locations (e.g., sensors 5 and 11; Fig 6B), sensor 14 was the only downstream benthic sensor to record low salinity values (~15 ppt) during low tide conditions. Thus, SGD distribution exhibited fine-scale variability, with groundwater remaining near the surface in some regions and extending to the benthos in others, driven by localized variations in reef geomorphology.

**Fig 7:**
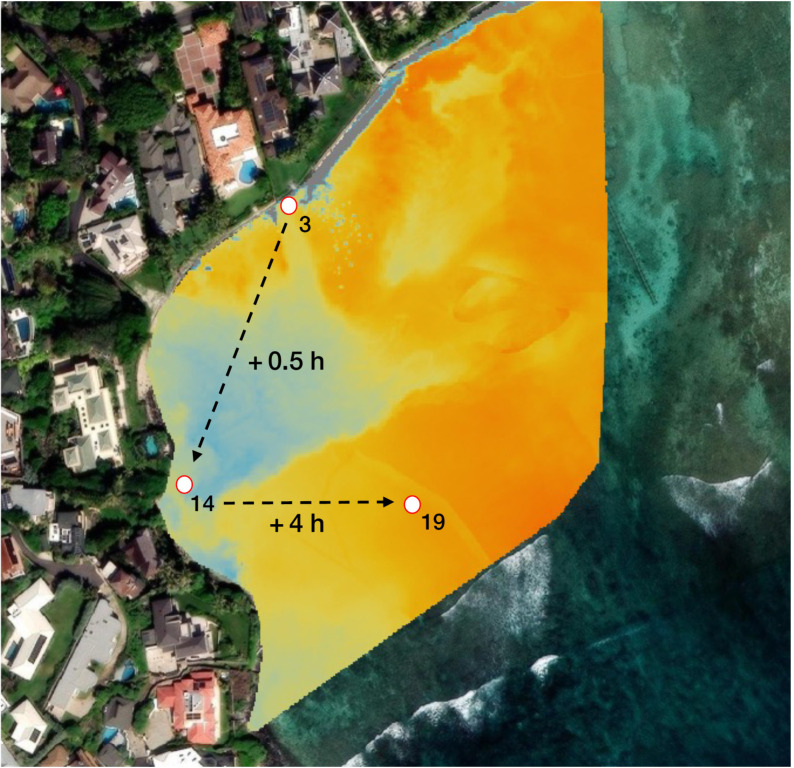
Cross-correlation between sensor 3 (seep) and sensor 14 (pooling area) showed a 24–48 minute lag in salinity response, with seep salinity changes preceding those downstream. Cross-correlation between sensor 14 and offshore sensor 19 indicated a 3.8–4 hour delay, consistent with observed salinity declines at sensor 19 preceding high tide. Arrows indicate temporal lags between sensors and represent a generalized direction of SGD movement during a rising tide; they do not capture all possible flow paths or hydrodynamic complexity. Created by authors using base maps from Esri. Content is the intellectual property of Esri and is used herein with permission. Copyright © 2025 Esri and its licensors. All rights reserved. Sources for basemaps: Resource Mapping Hawaii; Esri; Maxar/DigitalGlobe; Earthstar Geographics; CNES/Airbus DS; USDA FSA; USGS; Aerogrid; IGN; IGP; and the GIS User Community.

At sensor 3, located directly at the seep, salinity was highly correlated with tidal height (r = 0.920, p < 0.001), and cross-correlation analysis with tidal height showed salinity changes preceded tidal fluctuations by 18 minutes (lag = –3; r = 0.927; sampling frequency = 6 minutes). In contrast, sensor 14, positioned in an area where cooler water pooling was observed in thermal orthomosaics, exhibited a delayed salinity response. Cross-correlation between sensor 3 and 14 showed peak correlations (r ≈ 0.598–0.603) across lags 4–8, revealing that salinity changes at the seep preceded those at sensor 14 by approximately 24–48 minutes (Fig 7; S2A Fig). Thermal imagery supported these findings, showing persistent accumulation of cooler water near sensor 14 following low tide (Fig 5A–5D).

Cross-correlation between sensor 14 and offshore sensor 19 revealed peak correlations (r ≈ 0.616) across lags 38–40, corresponding to a 3.8–4 hour delay (Fig 7; S2B Fig). For example, on March 3, 2025, this lag placed salinity response at sensor 19 between 16:28 and 16:52, preceding high tide at 19:30. This timing aligns with the decrease in salinity at sensor 19, which began at 17:06 (Fig 6A). Thus, SGD pools along the western shoreline of the reef before being redistributed offshore via tidal advection. Autocorrelation results further reinforce this sequence: salinity at the seep remained autocorrelated for 3.3 hours, while sensors 14 and 19 exhibited persistent signals of 4.2 and 6.8 hours respectively (S2C–S2E Fig). Dynamic and tidally responsive discharge at the seep gives way to longer persistence times at the pooling zone, followed by the gradual dispersal of SGD into offshore waters.

## Discussion

### Fine-scale hydrodynamics drive spatial variability in SGD delivery

Our results demonstrate that localized hydrodynamics, in addition to tidal variability, further modulate SGD distribution heterogeneously across the reef, producing delayed patterns of groundwater delivery, retention, and offshore dispersal. CCF analysis revealed that salinity at sensor 3, located at the seep, exhibited an early response relative to tidal forcing, with salinity changes leading the tidal minimum by approximately 18 minutes. This early response is consistent with prior observations that SGD rates at Black Point increase during falling tide conditions as offshore hydrostatic pressure declines [19–21]. Additionally, salinity at sensor 14 exhibited a delayed response, lagging behind sensor 3 by approximately 24–48 minutes (Fig 7; S2A Fig). The delayed response at sensor 14 reflects the accumulation and pooling of groundwater on the surface following low tide, likely influenced by localized hydrodynamic conditions and reef structure. Lastly, a delayed low salinity signal was also observed at sensor 19, appearing approximately four hours after pooling was detected at sensor 14, indicating that accumulated groundwater is gradually transported offshore with the rising tide (Fig 6; S2B Fig).

Autocorrelation results further reinforce this sequence: dynamic and tidally responsive discharge at the seep gives way to longer persistence times at the pooling zone, followed by the gradual dispersal of SGD into offshore waters (S2C–S2E Fig). Specifically, the salinity signal at sensor 3 remained autocorrelated for approximately 3.3 hours while sensor 14 exhibited a longer persistence of 4.2 hours, consistent with SGD pooling observed in orthomosaics. Sensor 19 showed the most prolonged signal, 6.8 hours, demonstrating slow offshore transport. Similar patterns of localized temporal variability have been observed, with diffuse SGD discharge exhibiting distinct autocorrelation behaviors over small spatial scales that reflect heterogeneous groundwater dynamics [63]. Together, these results emphasize that SGD dynamics are not only driven by larger-scale tidal or wave processes but can also exhibit fine-scale temporal variability across different regions of the reef. Thus, time-series analyses are critical for capturing key variability periods in SGD distribution, accounting for the spatial and temporal complexity of groundwater delivery to coastal environments.

While our methods do not directly measure local hydrodynamics or residence times, our sUAS-TIR imagery and benthic salinity time series reveal persistent pooling in the western region of the reef, suggesting localized groundwater retention. We hypothesize that this pooling is driven by limited circulation, likely caused by reef geomorphology—specifically, a shallow patch of lava rocks that may restrict water exchange and prolong groundwater retention. Similar dynamics have been observed, with small-scale variations in reef geomorphology exerting a first-order control on flow patterns and residence times across reef systems [48]. These findings underscore that SGD influence is not uniformly distributed and cannot be conceptualized simply as a linear gradient from seep to offshore, where SGD concentration fades with distance from the seep. While the specific hydrodynamic drivers at Black Point are shaped by local geomorphology, the methodological framework presented here remains broadly applicable for identifying spatial sensitivity to SGD in other groundwater impacted environments.

The spatial heterogeneity of SGD delivery, shaped by both tidal forcing and local hydrodynamics, strongly influences benthic community structure. At Black Point, areas experiencing sustained SGD influence are dominated by invasive and opportunistic algal species [16], which outcompete other producers and reduce overall community diversity. Such community shifts are likely driven by elevated nutrient concentrations and altered carbonate chemistry associated with SGD, including reduced carbonate and bicarbonate ion availability [6]. As a result, areas of strong SGD influence have a limited distribution of calcifying algal species [16], further promoting conditions that favor the proliferation of opportunistic fleshy algae. SGD also imposes salinity stress on corals, with documented declines in growth, survivorship, and calcification rates near the seep at Black Point [6,13]. Notably, recent analyses have shown that both semi-diurnal tidal and diel-scale SGD variability are significantly correlated with spatial variation in community composition at Black Point [65], reinforcing the role of fine-scale SGD delivery dynamics in structuring benthic assemblages. Our findings further reveal that SGD dispersal is both spatially and temporally heterogeneous, with certain reef zones experiencing prolonged retention. These pooling areas are subject to sustained nutrient enrichment and reduced flushing, creating localized conditions of elevated ecological sensitivity. Benthic producers in these areas may experience intensified stress, including increased mortality [48,49], reduced growth and productivity [13,50,66–68], and greater susceptibility to disease [51–54]. Thus, understanding the ecological implications of SGD depends not only on quantifying the presence and concentration of low-salinity, nutrient-rich groundwater, but also elucidating the local mixing conditions that govern SGD-influence on the benthos and water column. By resolving these fine-scale patterns, our drone-based thermal imagery and benthic salinity time-series approach offers a valuable tool for identifying specific areas where SGD may strongly, and disproportionately, influence community structure. These insights are critical for informing conservation strategies, as effective management must account for spatially variable SGD impacts.

### Multi-scale analysis of SGD distribution

**Fig 8:**
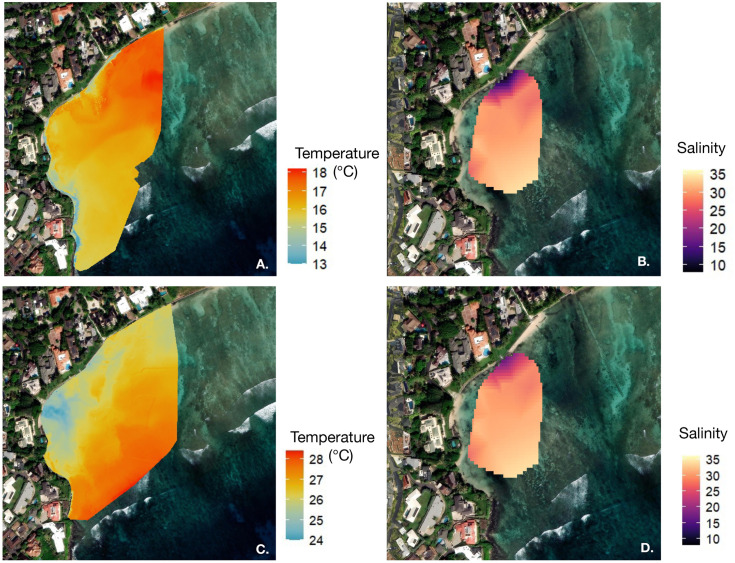
Surface thermal orthomosaics (A, C) collected on 1 March 2025 and benthic salinity maps (B, D) collected on 2 March 2025, all following low tide. The top and bottom rows represent tidal heights of 0.08 m and 0.24 m, respectively. Thermal imagery captured a broader spatial extent of the SGD plume and offshore dispersal than benthic salinity maps, which failed to resolve a large mid-reef portion of the plume and appeared relatively static over a two-hour period. Created by authors using base maps from Esri. Content is the intellectual property of Esri and is used herein with permission. Copyright © 2025 Esri and its licensors. All rights reserved. Sources for basemaps: Resource Mapping Hawaii; Esri; Maxar/DigitalGlobe; Earthstar Geographics; CNES/Airbus DS; USDA FSA; USGS; Aerogrid; IGN; IGP; and the GIS User Community.

Thermal orthomosaics were collected on 1 March 2025 (Fig 8A, 8C) and benthic salinity maps were collected on 2 March 2025 (Fig 8B, 8D). All maps were collected after low tide, with the top and bottom rows representing tidal heights of 0.08 m and 0.24 m, respectively. Compared to benthic salinity maps, surface thermal imagery captured a broader spatial extent of the SGD plume and clearly depicted offshore SGD dispersal. Although key features such as the seep, western pooling zone, and near-seep SGD distribution are identifiable in the benthic maps, a large mid-reef portion of the plume is absent, and offshore mixing is poorly resolved. These differences indicate that in some areas of the reef, SGD remains buoyant at the surface rather than reaching the benthos. This stratification is primarily driven by salinity induced density differences, which limit the downward transport of fresher groundwater [7,35]. Bathymetric features, such as high relief, can further influence SGD flow paths and retention across reefs [7,48]. Consistent with these patterns, our benthic salinity sensor data showed weaker freshwater signatures in mid-reef areas, despite the presence of a surface plume evident in the thermal imagery. Additionally, while the pooling region is distinguishable by its lower salinity signature, the two benthic salinity maps appear relatively static over a two-hour period, failing to capture the progressive accumulation of SGD following low tide conditions.

Although benthic salinity maps provided limited spatial resolution of plume dynamics, time-series analysis of benthic salinity data revealed clear temporal patterns of SGD delivery, retention, and delayed offshore mixing. These results highlight the value of a two-pronged mapping approach, integrating both drone-based surface thermal imagery and benthic salinity time series to capture fine-scale SGD dynamics. While surface thermal imagery provides real-time visualization of mixing dynamics, broader spatial coverage, and flexible, repeated sampling, the fine-scale temporal resolution provided by benthic sensors is critical for characterizing lagged salinity responses and tracking water movement across tidal cycles.

Furthermore, the mixing dynamics of SGD emphasize the need for an integrated methodology. Because SGD is fresher, and therefore less dense than ambient seawater, it remains buoyant on the surface before mixing vertically [35,36]. As a result, the extent of SGD is greater at the surface compared to the benthos, where groundwater signatures are less distinct. Moreover, coastal mixing dynamics are driven by changing environmental conditions (e.g., bathymetry, wind, waves, substrate composition, etc.), affecting surface and benthic water masses to different extents [48,69]. Given the dynamic nature of these near-shore environments, a combined assessment of surface and benthic properties is essential to accurately characterize SGD distribution and impacts across both spatial and temporal scales.

### Applications of sUAS-TIR systems for SGD mapping

This study demonstrates the applicability of using small unmanned aerial systems equipped with thermal infrared sensors to map the spatial distribution of submarine groundwater discharge on shallow coral reefs. The high spatial resolution (~15–25 cm) of our thermal imagery allowed for detailed visualization of cooler SGD plumes, even in a dynamic and tidally influenced nearshore environment. This approach provides a high-resolution alternative to traditional in situ point sampling methods (e.g., geochemical tracers, piezometers), which can be labor-intensive and spatially constrained [2,29,33].

The use of sUAS platforms has greatly improved temporal sampling resolution compared to manned aircraft systems, as they can be deployed directly from field sites and are significantly less expensive to operate [32]. Building on this advancement, our study highlights the functionality of a DJI Enterprise drone system for thermal mapping of SGD. Whereas previous studies often relied on customized sUAS-TIR systems that required independent integration of drone, TIR-sensor, and visual-sensor components [32,45–47], our methods utilized a cost-effective, fully integrated system that did not require additional modification. This lower-cost, user-friendly system enabled high-frequency thermal mapping essential for capturing SGD variability driven by tides and local circulation and offers an accessible approach well-suited for future place-based science initiatives and community collaborations.

Moreover, the flexibility of the sUAS platform allows for real-time observation of coastal oceanographic processes, by capturing the spatial and temporal variability of SGD mixing (see video in Supporting Information). Image resolution can be controlled by adjusting flight altitude, enabling either high-resolution imagery at lower altitudes or broader coverage at higher altitudes, depending on research objectives and site conditions [32]. Live thermal imagery transmitted during flight offers the potential for adaptive targeting, identification of new SGD sources, and detection of dynamic circulation patterns that might otherwise go unobserved. By observing both surface SGD signatures and circulation patterns in real time, we identified that the SGD plume at Black Point was more extensive than previously understood. While earlier studies had documented SGD at this site [5,16], their sampling did not include the western shoreline of the reef, where our thermal imagery revealed patterns indicative of groundwater pooling. In response to these observations, visible both in the orthomosaics and in real-time thermal imagery, we expanded our benthic sensor grid to include this region, confirming that the colder water pooling in TIR imagery was SGD. This ability to rapidly identify and target regions heavily impacted by SGD highlights the value of drone-based thermal imagery, enabling researchers to refine sampling strategies in real time.

### Limitations, future directions, and conclusions

While the combined use of surface thermal imagery and benthic salinity time series offers valuable insights into SGD dynamics, certain limitations should be considered. Though suited for remote deployment, drone operations are limited by environmental conditions, including rain and strong winds. Even moderate wind can influence surface water mixing, potentially altering the structure and dispersion of the SGD plume [70,71]. Furthermore, seasonal differences affect plume detectability, with stronger thermal contrasts in warmer months enhancing plume visibility. Seasonal variation in wave action and wind exposure also influences plume behavior, with higher hydrodynamic energy leading to increased water column mixing [72,73]. Although flight altitude allows control over image resolution, there are trade-offs between resolution, battery life, and mapping efficiency. Despite increasing image resolution, lower, longer flights lead to greater variability in orthomosaic stitching. While key features of the SGD plume were consistently and accurately captured, orthomosaic production relies on distinct identifiable structures for alignment, making mid-reef areas with uniform water surfaces difficult to map. Although our TIR-sUAS system accurately captured the spatial distribution of SGD, its ± 2 °C temperature accuracy limits its suitability for precise surface temperature measurements. However, for our objective, mapping relative thermal patterns associated with SGD, a fully integrated, user-friendly system was ideal, particularly for developing methods suitable for future community-based applications. Despite these limitations, when carefully implemented with consideration of environmental conditions, survey design, and post-processing choices, the combined use of sUAS-TIR imagery and benthic salinity sensors remains a powerful and effective approach for characterizing dynamic SGD patterns across coastal reef environments.

Through the integration of drone-based infrared imagery with benthic salinity time series, we elucidated fine-scale SGD dynamics that were previously unobservable, demonstrating that localized hydrodynamics strongly modulate groundwater distribution. This two-pronged approach revealed complex patterns of SGD dispersion associated with ecological vulnerability, including areas of prolonged exposure to nutrient-rich, low-salinity waters. By offering a scalable and operationally flexible framework, our methodology enables the identification of reef zones most susceptible to SGD-driven stressors, with broad applicability for the management of groundwater-impacted ecosystems. Similarly, future work can leverage this operational flexibility to investigate how environmental factors (e.g., reef bathymetry, substrate composition, wind-driven currents, post-rainfall events) influence SGD dispersion and coastal mixing at fine spatial and temporal scales. To fully understand the influence of SGD on reef ecosystems, it is essential to account for the spatial and temporal variability in exposure, recognizing that biogeochemical processes and biological responses are shaped by dynamic, heterogeneous patterns of groundwater delivery and retention. As SGD continues to affect coastal ecosystems globally, such integrative approaches are critical for evaluating groundwater-driven ecological change and guiding effective conservation strategies.

## Supporting information

S1 Table Repeated surveys conducted between May and June 2024 showed consistent plume size and orientation at low tide (0.04 m), despite variable wind conditions. Wind speed from NOAA CDO station WBAN:22521.
(DOCX)

S1 Fig Thermal orthomosaics from repeated surveys on (A) May 13, (B) June 11, (C) June 12, and (D) June 14, 2024 showed a consistently oriented plume at low tide (0.04 m), underscoring persistent discharge dynamics, stable plume behavior across short temporal scales (i.e., weeks to months), and consistent orthomosaic production despite variable wind conditions. (TIF)

S2 Fig (**A**) **Cross-correlation between sensor 3 (seep) and sensor 14 (pooling area) showed a 24–48 minute lag in salinity response, with seep salinity changes preceding those downstream.** (**B**) Cross-correlation between sensor 14 and offshore sensor 19 indicated a 3.8–4 hour delay, consistent with observed salinity declines at sensor 19 preceding high tide. (**C–E**) Autocorrelation analyses revealed persistent salinity signals of 3.3 hours at the seep, 4.2 hours at the pooling zone, and 6.8 hours offshore, further reinforcing this temporal sequence.
(TIF)

S1 Video Live TIR imagery of the seep at low tide illustrates fine-scale spatial variability in SGD delivery at sub-meter scales (~15–25 cm). Cooler surface temperatures are dark purple, while yellow indicates warmer sea surface temperatures.
(ZIP)
