# Supplementary material for: Drone thermal imaging and benthic time-series analysis show dynamic spatial and temporal delivery of submarine groundwater discharge on reef ecosystems
Source: PLoS One. 2025 Oct 3;20(10):e0333712. doi: 10.1371/journal.pone.0333712 (PMC12494286; doi:10.1371/journal.pone.0333712)
Supplement: S1 Table — Wind speed from NOAA CDO station WBAN:22521. (DOCX) [file pone.0333712.s001.docx]

**Supporting Information**

**S1 Table.** Repeated surveys conducted between May and June 2024 showed consistent plume size and orientation at low tide (0.04 m), despite variable wind conditions. Wind speed from NOAA CDO station WBAN:22521.

| Date | Time Since Low Tide | Wind Speed | Approximate Surface Area |
| --- | --- | --- | --- |
| 2024-05-13 | 72 minutes | 5.82 m/s | 8,334 m² |
| 2024-06-11 | 62 minutes | 13.42 m/s | 8,174.82 m² |
| 2024-06-12 | 73 minutes | 11.41 m/s | 8,343.34 m² |
| 2024-06-14 | 47 minutes | 14.32 m/s | 8,395.12 m² |
