# Supplementary figures and images for: Drone thermal imaging and benthic time-series analysis show dynamic spatial and temporal delivery of submarine groundwater discharge on reef ecosystems
Source: PLoS One. 2025 Oct 3;20(10):e0333712. doi: 10.1371/journal.pone.0333712 (PMC12494286; doi:10.1371/journal.pone.0333712)

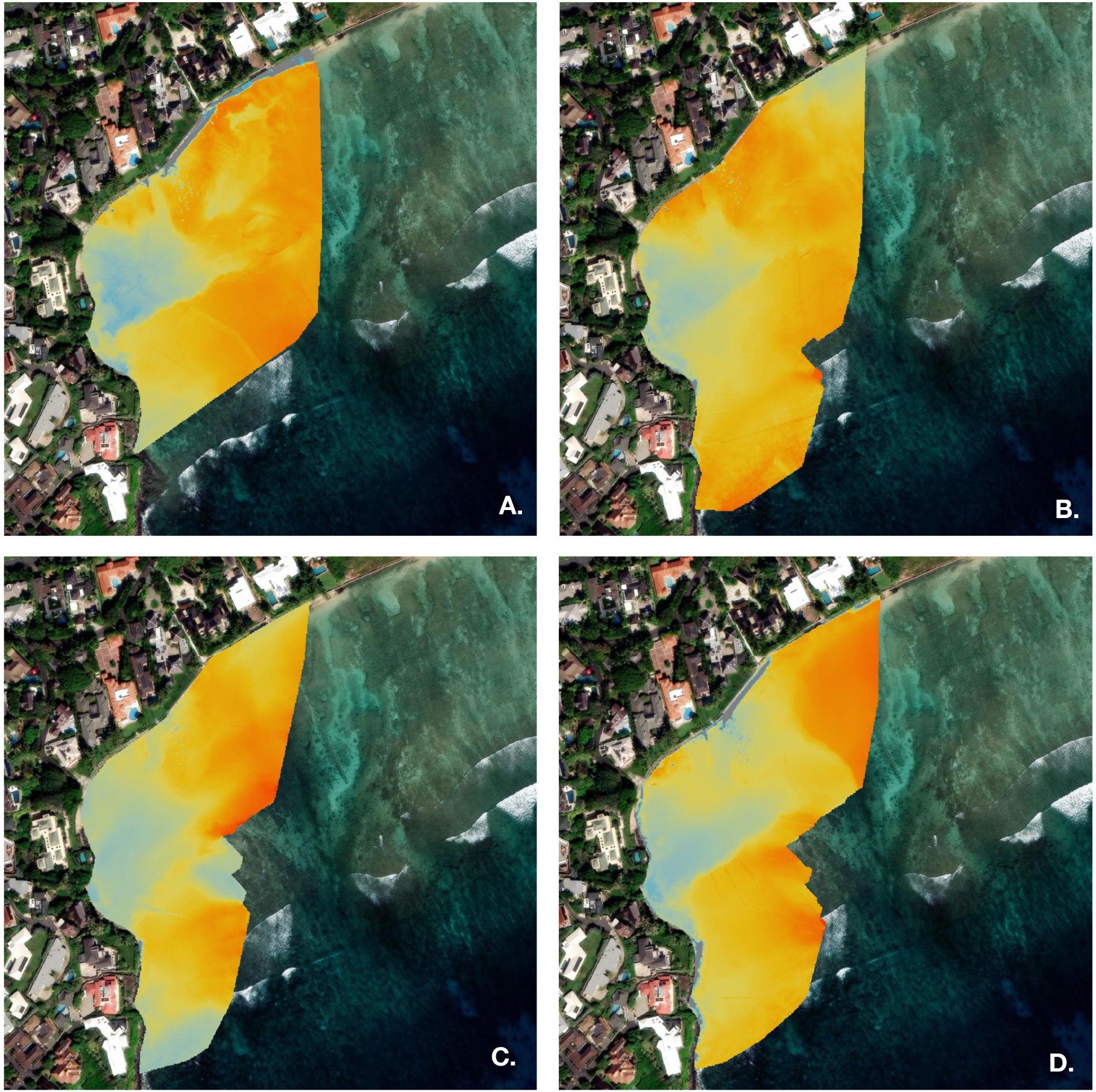

Supplement: S1 Fig — (TIF) [file pone.0333712.s002.tif]

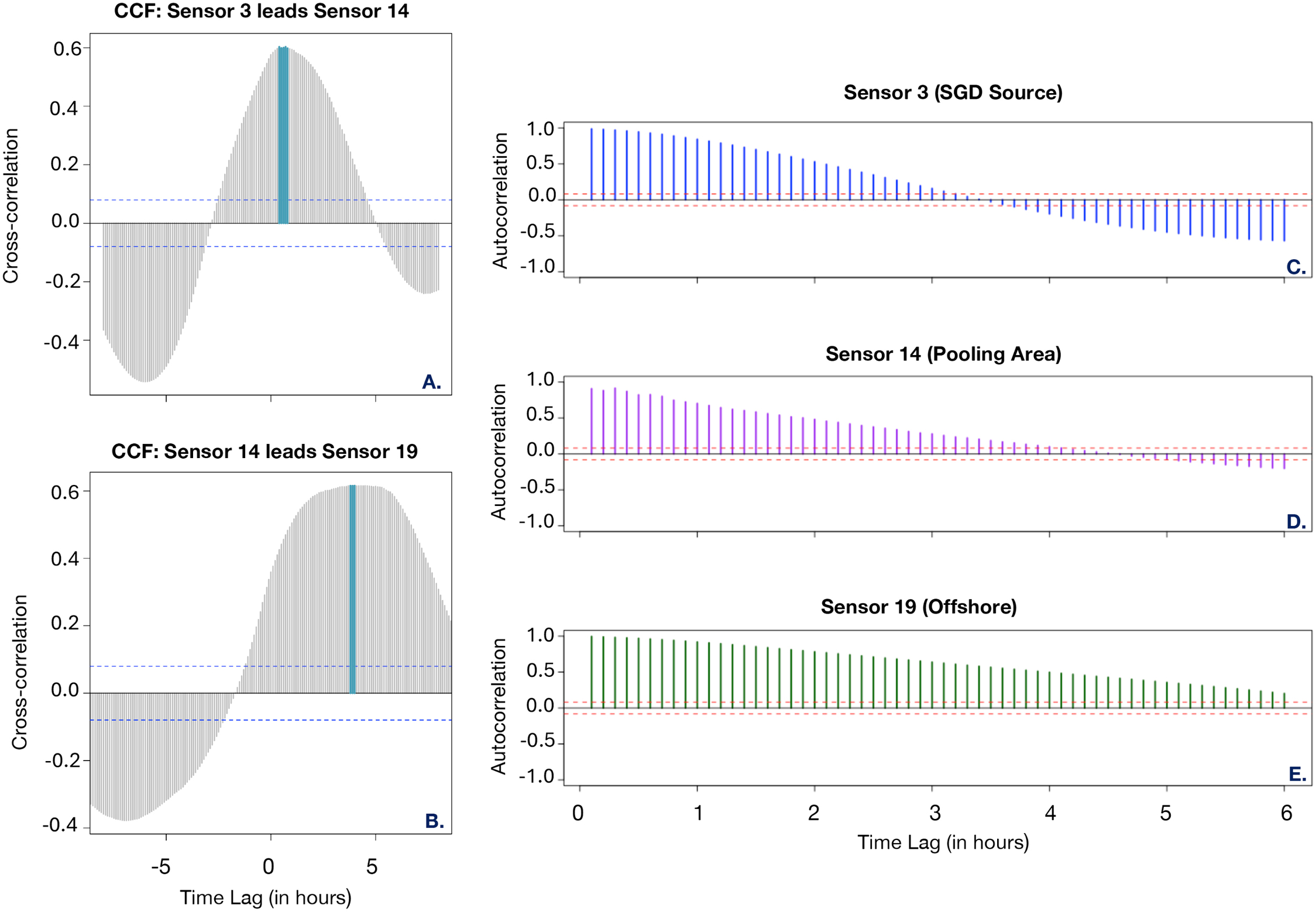

Supplement: S2 Fig — (A) Cross-correlation between sensor 3 (seep) and sensor 14 (pooling area) showed a 24–48 minute lag in salinity response, with seep salinity changes preceding those downstream. (B) Cross-correlation between sensor 14 and offshore sensor 19 indicated a 3.8–4 hour delay, consistent with observed salinity declines at sensor 19 preceding high tide. (C–E) Autocorrelation analyses revealed persistent salinity signals of 3.3 hours at the seep, 4.2 hours at the pooling zone, and 6.8 hours offshore, further reinforcing this temporal sequence. (TIF) [file pone.0333712.s003.tif]
